# lncRNA profile study reveals the mRNAs and lncRNAs associated with docetaxel resistance in breast cancer cells

**DOI:** 10.1038/s41598-018-36231-4

**Published:** 2018-12-19

**Authors:** Peide Huang, Fengyu Li, Lin Li, Yuling You, Shizhi Luo, Zhensheng Dong, Qiang Gao, Song Wu, Nils Brünner, Jan Stenvang

**Affiliations:** 1 Section of Pharmacotherapy, Department of Drug Design and Pharmacology, Faculty of Health and Medical Sciences, University of Copenhagen, 2200 Copenhagen N, Denmark; 2 The Affiliated Luohu Hospital of Shenzhen University, Shenzhen Luohu Hospital Group, Shenzhen, China; 3 BGI Genomics, BGI-Shenzhen, Shenzhen, 518083 China; 4 The First Affiliated Hospital of Shenzhen University, Shenzhen, China

## Abstract

Resistance to adjuvant systemic treatment, including taxanes (docetaxel and paclitaxel) is a major clinical problem for breast cancer patients. lncRNAs (long non-coding RNAs) are non-coding transcripts, which have recently emerged as important players in a variety of biological processes, including cancer development and chemotherapy resistance. However, the contribution of lncRNAs to docetaxel resistance in breast cancer and the relationship between lncRNAs and taxane-resistance genes are still unclear. Here, we performed comprehensive RNA sequencing and analyses on two docetaxel-resistant breast cancer cell lines (MCF7-RES and MDA-RES) and their docetaxel-sensitive parental cell lines. We identified protein coding genes and pathways that may contribute to docetaxel resistance. More importantly, we identified lncRNAs that were consistently up-regulated or down-regulated in both the MCF7-RES and MDA-RES cells. The co-expression network and location analyses pinpointed four overexpressed lncRNAs located within or near the *ABCB1* (ATP-binding cassette subfamily B member 1) locus, which might up-regulate the expression of *ABCB1*. We also identified the lncRNA EPB41L4A-AS2 (EPB41L4A Antisense RNA 2) as a potential biomarker for docetaxel sensitivity. These findings have improved our understanding of the mechanisms underlying docetaxel resistance in breast cancer and have provided potential biomarkers to predict the response to docetaxel in breast cancer patients.

## Introduction

Breast cancer is the most frequently diagnosed cancer among females and the leading cause of cancer death among women worldwide^1^. Multiple treatment modalities are used in breast cancer, such as surgery, radiation therapy, chemotherapy and target therapy. Taxanes, including docetaxel and paclitaxel, are among the most commonly used chemotherapeutic agents to treat breast cancer^2^. However, patients receiving a taxane treatment may become resistant to taxanes, thus resulting in recurrence and metastatic disease.

To reduce patient suffering from disease recurrence caused by taxane resistance, researchers focus on the molecular mechanisms and predictive biomarkers of taxane resistance at multiple levels. Several genes are associated with taxane resistance. The differential expression of the *ABCB1* gene is one of the most studied putative biomarkers in taxane-resistant cancers^3,4^. Pgp (permeability glycoprotein), encoded by the *ABCB1* gene, has been reported to act as an ATP-dependent efflux pump and reduce taxane concentration by expelling the drug^5^. Other genes, such as *ABCG2* (ATP binding cassette subfamily G member 2)^6^ *ABCB4* (ATP binding cassette subfamily B member 4)^7^ are also involved in the process of taxane resistance. However, the underlying mechanism of taxane resistance in breast cancer is still not fully elucidated, and the regulators of the taxane-resistant genes remain unknown. As a result, there are presently no predictive biomarkers for taxanes in clinical use.

Long non-coding RNAs (lncRNAs) are defined as RNAs longer than 200 nucleotides, with little potential in protein coding. Many lncRNAs are described to influence mRNA generation and expression^8^. Recent studies have shown that lncRNAs are also implicated in chemotherapy resistance. The lncRNA HOTAIR (HOX Transcript Antisense RNA) is reported to contribute to cisplatin resistance in human lung adenocarcinoma cells via down-regulating p21^WAF1/CIP1^ expression^9^. Another study found that the lncRNA MRUL (multidrug resistance related and up-regulated lncRNA) promotes *ABCB1* expression in multidrug-resistant gastric cancer^10^. More recently, data have shown that two lncRNAs, ROR (regulator of reprogramming) and CCAT1 (colon cancer-associated transcript-1), regulate docetaxel resistance in lung adenocarcinoma^11,12^. Furthermore, from a transcriptome microarray study, the lncRNAs HIF1A-AS2 (HIF1A Antisense RNA 2) and AK124454 were shown to promote cell proliferation and invasion in TNBC (triple-negative breast cancers) cells and contribute to paclitaxel resistance^13^. However, the contribution of lncRNAs to docetaxel resistance in breast cancer is still unclear.

In this study, we carried out whole transcriptome sequencing in two cell lines, MCF-7 and MDA-MB-231, and in their docetaxel-resistant sublines, MCF7-RES and MDA-RES. We identified significantly differentially expressed (SDE) mRNAs and lncRNAs between the parental and resistant sublines, and we also uncovered the potential relationship between the SDE mRNAs and lncRNAs. Compared with previous studies, we discovered several novel genes in addition to *ABCB1* which might contribute to the taxane-resistant phenotype of breast cancers. More important, we identified a group of lncRNAs that might potentially regulate taxane sensitivity by controlling the expression of chemotherapy-resistant genes.

## Results

### Sequencing results and quality control

By performing Illumina-based RNA-Seq sequencing, a total of 1,825,984,984 raw reads were produced from the 12 RNA samples (3 independent samples from each cell line (Table 1)). After quality control, 1,750,124,272 clean reads (157.5 Gb) were obtained.

**Table 1:** Summary of data yield and quality control.

Sample	MCF7_12_P	MCF7_14_P	MCF7_15_P	MCF7_13_T	MCF7_14_T	MCF7_15_T	MDA_13_P	MDA_14_P	MDA_16_P	MDA_14_T	MDA_15_T	MDA_16_T
**Number of raw reads**	155,368,324	150,086,362	154,603,830	151,890,702	146,565,380	150,531,888	154,601,446	150,960,982	155,560,554	150,067,038	154,602,638	151,145,840
**Raw data size(Gb)**	13.98	13.51	13.91	13.67	13.19	13.55	13.91	13.59	14.00	13.51	13.91	13.60
**Number of clean reads**	147,254,586	145,503,292	148,444,124	145,691,082	141,393,020	143,013,044	149,175,500	146,265,718	146,883,552	144,846,612	149,085,610	142,568,132
**Clean data size(Gb)**	13.25	13.10	13.36	13.11	12.73	12.87	13.43	13.16	13.22	13.04	13.42	12.83
**Clean data/Raw data(%)**	94.78	96.95	96.02	95.92	96.47	95.01	96.49	96.89	94.42	96.52	96.43	94.32
**Q30 reads(%)**	88.66	92.75	89.63	89.73	92.72	88.81	90.36	92.57	89.23	92.96	90.54	88.38
**GC(%)**	49.87	47.26	49.83	49.46	47.33	49.89	47.54	46.84	47.42	46.58	48.24	49.61
**Reads mapped to genome**	126,241,357	129,177,823	134,000,511	133,059,665	125,613,559	122,233,249	133,556,825	129,562,173	126,202,348	128,884,515	132,119,668	118,246,009
**Mapping rate(%)**	85.73	88.78	90.27	91.33	88.84	85.47	89.53	88.58	85.92	88.98	88.62	82.94

For the sample names, P represents the parental cells, T represents the docetaxel-resistant cells, number represents the passage.

The proportion of clean reads among the raw reads of the 12 samples ranged from 94.32% to 96.95%. The proportion of clean reads with a Phred quality value greater than 30 among the samples ranged from 88.38% to 92.96%. The GC (guanine and cytosine nucleotides) content of the clean reads of the 12 samples ranged from 47.26% to 49.89%. In total, 82.94% to 91.33% of the clean reads were aligned against the human reference genome (Table 1).

### Overview of mRNA expression and the significantly differentially expressed mRNAs between the parental and docetaxel-resistant cell groups

A total of 13,714 mRNAs were detected. More than 97.81% of the mRNAs was detected simultaneously among 3 passages of the same cell line (Sup Fig. 1A). The FPKM (fragments per kilobase million) values of the 13,714 mRNAs from 12 samples were centralized and normalized for the PCA (Principal component analysis). The results show that PC1 (Principal component 1) accounted for 52.28% of the total variance and could separate the MDA cells from the MCF7 cells; PC2 (Principal component 2) accounted for 13.91% of the total variance and separated the parental and docetaxel-resistant MDA cells. However, parental and docetaxel-resistant MCF7 cells clustered together (Sup Fig. 1B).

**Figure 1:**
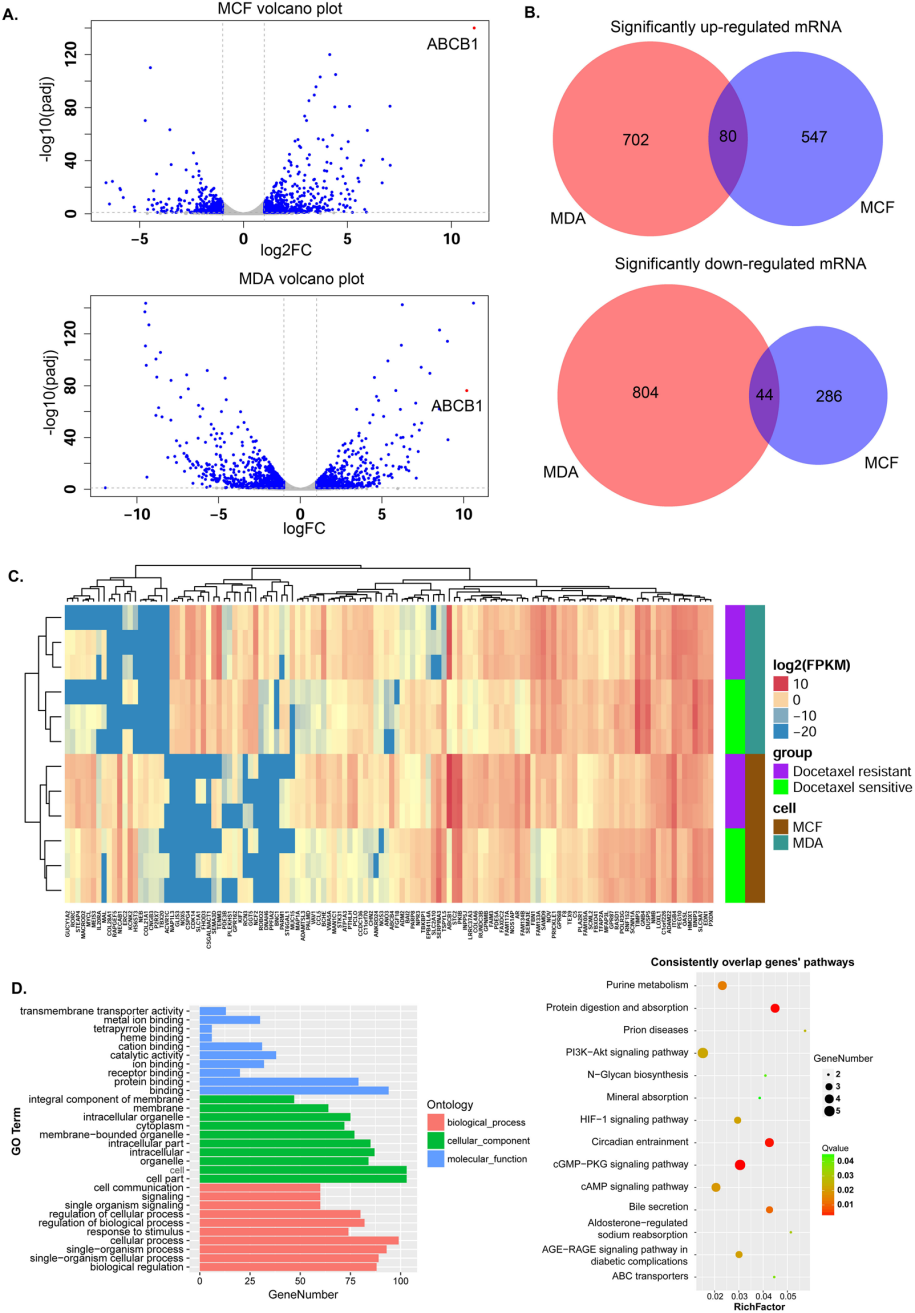
Overview of the mRNA expression and significantly differentially expressed mRNAs between the parental and docetaxel-resistant cell groups. (**A**) Volcano figure showing the significantly differentially expressed genes in the MDA-RES and MCF7-RES cells in comparison to their parental cells. The blue dots represent mRNAs with |Log2FC| > 1, FDR < 0.1. (**B**) Venn diagrams of the significantly up-regulated or down-regulated mRNAs in both of the cells. (**C**) Complete-linkage clustering with the 124 consistent SDE mRNAs in the 12 samples. D. KEGG pathway enrichment and GO analysis for the consistent SDE genes. Left panel: GO functional classification of the consistent SDE genes. Red, green, and blue represent the three classes of the GO term. The top 10 enriched GO terms are shown in each class. Right panel: Scatter plot for the KEGG enrichment of the consistent SDE genes. RichFactor is the ratio of the consistent SDE genes annotated in a pathway to all the genes in this pathway. The Q value is the corrected p-value. The top 14 pathways with a Q value < 0.05 are shown in the figure.

Differentially expressed mRNAs between the parental and docetaxel-resistant cell groups were calculated by DESeq in the two cell lines. The criteria of |Log_2_FC| > 1 (Log_2_ (fold change)), FDR < 0.1 (false discovery rate) was used to select the SDE mRNAs. We identified 1630 and 957 SDE mRNAs in the MDA-RES and MCF7-RES groups by comparing them to their parental groups, respectively, as shown in the volcano figure (Fig. 1A).

In the MDA-RES cells, 782 mRNAs were significantly up-regulated, and 848 mRNAs were significantly down-regulated. In the MCF7-RES cells, 627 mRNAs were significantly up-regulated, and 330 mRNAs were significantly down-regulated.

The most studied MDR (multidrug resistance) related gene, *ABCB1* was dramatically up-regulated in both the MDA-RES and MCF7-RES cells (Fig. 1A), which was consistent with a previous report^4^. The top ten significantly up- or down-regulated genes in the MDA-RES or MCF7-RES cells are shown in Sup Table 1.

By analyzing the list of significantly up-regulated and down-regulated mRNAs in the MDA-RES and MCF7-RES cells, we found 80 mRNAs that were consistently up-regulated and 44 mRNAs that were consistently down-regulated in both of the cells (Fig. 1B, Sup Table 2).

Using these 124 consistent SDE mRNAs, the 12 samples were clustered into two groups (MDA-MB-231 and MCF-7) (Fig. 1C), which indicated that the mRNA diversity between the MCF-7 cells and MDA-MB-231 cells was greater than that between the docetaxel-resistant and parental cells.

### GO (Gene ontology) and KEGG (Kyoto Encyclopedia of Genes and Genomes) pathway analyses of the significantly differentially expressed genes

A KEGG pathway analysis was performed in each cell line. In the MCF7-RES cells, the three most enriched KEGG pathways associated with the SDE mRNAs were the metabolic pathways, axon guidance, and neuroactive ligand-receptor interaction. However, other pathways, such as the PI3K-Akt signaling pathway, Wnt signaling pathway, ECM-receptor interaction and ABC transporters, were also significantly enriched (Sup Fig. 2).

In the MDA-RES cells, the PI3K-Akt signaling pathway, cytokine-cytokine receptor interaction, and pathways in cancer were the top three pathways, followed by the metabolic pathway, AGE-RAGE signaling pathway, and the cell adhesion molecules (CAMs) pathway. Furthermore, ABC transporters, the MAPK signaling pathway and ECM-receptor interaction were also significantly enriched (Sup Fig. 3).

We also performed the KEGG pathway analysis on the 124 consistent SDE mRNAs shared by the MCF7 and MDA cells. As shown in Fig. 1D, 14 signaling pathways were significantly enriched (Q value < 0.05). The most significantly enriched pathway was the cGMP-PKG signaling pathway. However, the ABC transporters, PI3K-Akt signaling pathway, AGE-RAGE signaling pathway in diabetic complications, and HIF-1 signaling pathway were also significantly enriched.

A GO analysis for the consistent SDE mRNAs was also performed to identify the function of the coding transcripts. With regard to biological processes, the most enriched GO term associated with the consistent SDE mRNAs was cellular process. With regard to the cellular component, the most enriched GO terms were cell and cell part. Moreover, the most enriched GO terms associated with the consistent SDE mRNAs for molecular function were binding and protein binding (Fig. 1D).

### Overview of lncRNA expression and the significantly differentially expressed lncRNAs between the parental and docetaxel-resistant cell groups

**Figure 2:**
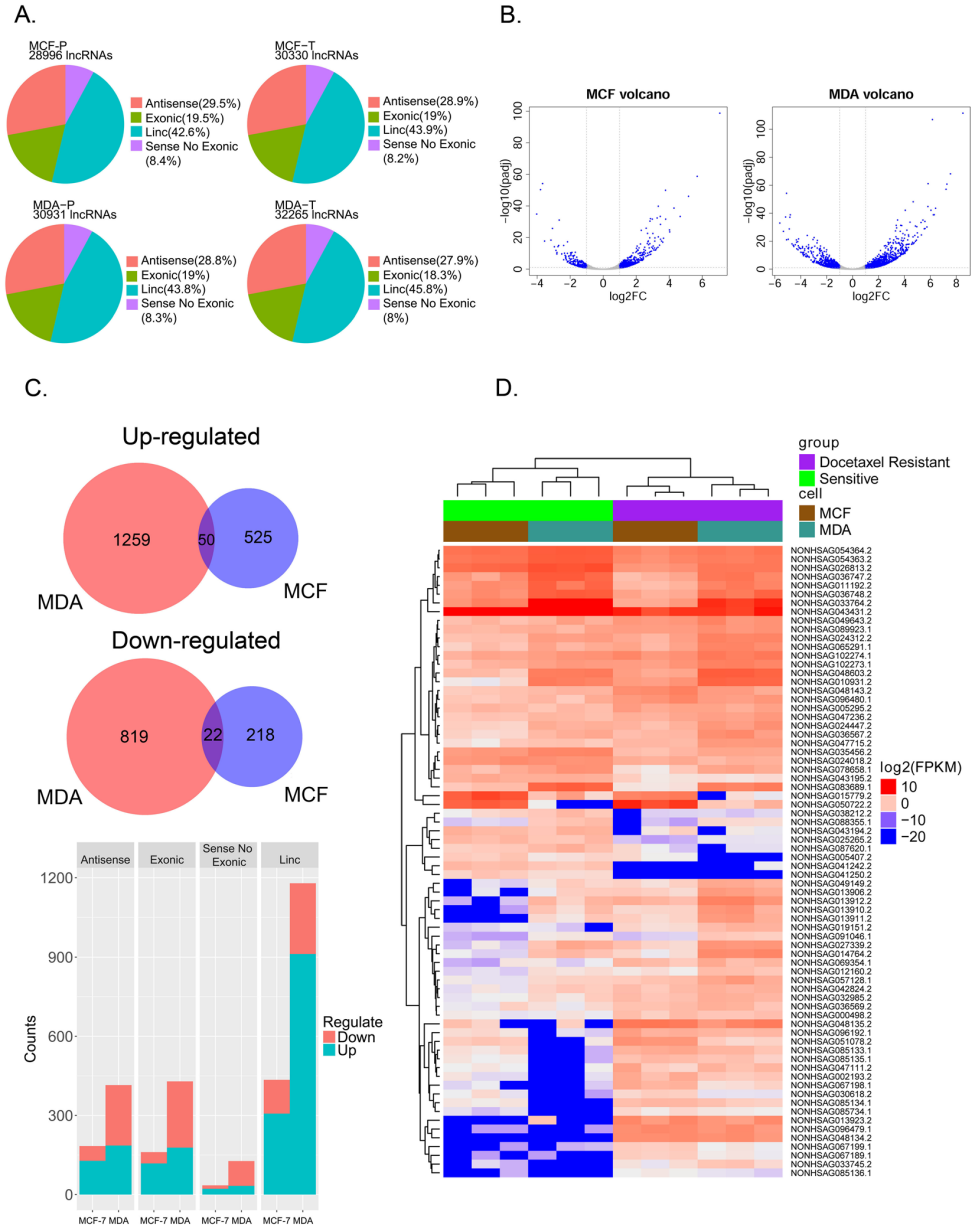
Overview of the lncRNA expression and significantly differentially expressed lncRNAs between the parental and docetaxel-resistant cell groups. (**A**) The subgroups and number of lncRNAs detected in the docetaxel-resistant and parental cell lines (P represents the parental cell line; T represents the docetaxel-resistant cell line). (**B**) Volcano figure showing the significantly differentially expressed lncRNAs in the MDA-RES and MCF7-RES cells compared to their parental cells. The blue dots represent lncRNAs with |Log2FC| > 1, FDR < 0.1. (**C**) Upper panel: Venn diagrams of consistently up-regulated or down-regulated lncRNAs in both of the MDA-RES and MCF7-RES cells. Lower panel: the bar chat shows the subgroups and numbers of significantly up-regulated or down-regulated lncRNAs. (**D**) Complete-linkage clustering with the 72 consistent SDE lncRNAs in the 12 samples.

NONCODE 2016 was used to annotate the lncRNAs. The lncRNAs were classified into 4 subgroups as antisense, exonic, linc (long intergenic non-coding), and sense no exonic lncRNAs, according to their location and their relationship with nearby genes. The classification of the lncRNAs in the NONCODE database was provided online (http://www.noncode.org/datadownload/NONCODE2016_human_cc.tgz). We found that lincRNAs and antisense lncRNAs were the two most abundant subtypes in all the cell lines (Fig. 2A).

A total of 39,363 lncRNAs were identified in the 12 RNA samples. More than 81.55% of the lncRNAs were detected simultaneously among three independent passages of the same cell line (Sup Fig. 3A). The differentially expressed lncRNAs between the parental and docetaxel-resistant cell groups were calculated by DESeq. We identified 2,150 and 815 SDE lncRNAs (|Log2FC| > 1, FDR < 0.1) in MDA-RES and MCF7-RES groups (Fig. 2B,C). The SDE lncRNAs identified in the MDA-RES cells included 415 antisense lncRNAs, 1179 intergenic lncRNAs, 429 exonic lncRNAs and 127 sense no exonic lncRNAs. While in the MCF7-RES cells, the SDE lncRNAs included 184 antisense lncRNAs, 435 intergenic lncRNAs, 161 exonic lncRNAs, and 35 sense no exonic lncRNAs (Fig. 2C).

By analyzing the list of SDE lncRNAs in the MCF7-RES and MDA-RES cells, we found 50 lncRNAs that were consistently up-regulated, while 22 lncRNAs were consistently down-regulated in both the MCF7-RES and MDA-RES cell lines (Fig. 2C, Sup Table 3).

By performing complete-linkage clustering with these consistent SDE lncRNAs, the 12 samples were clustered into two groups (parental and resistant) perfectly (Fig. 2D).

### lncRNA-mRNA co-expression network construction and docetaxel-resistant module detection

It is well documented that lncRNAs function as regulators of target mRNAs^8,14^. Additionally, lncRNAs and their target mRNAs usually present similar expression patterns^15–17^. To predict the target mRNAs of the lncRNAs, we used the WGCNA (weighted gene co-expression network analysis)^18^ R software package to detect similar expression patterns between the SDE lncRNAs and mRNAs.

**Figure 3:**
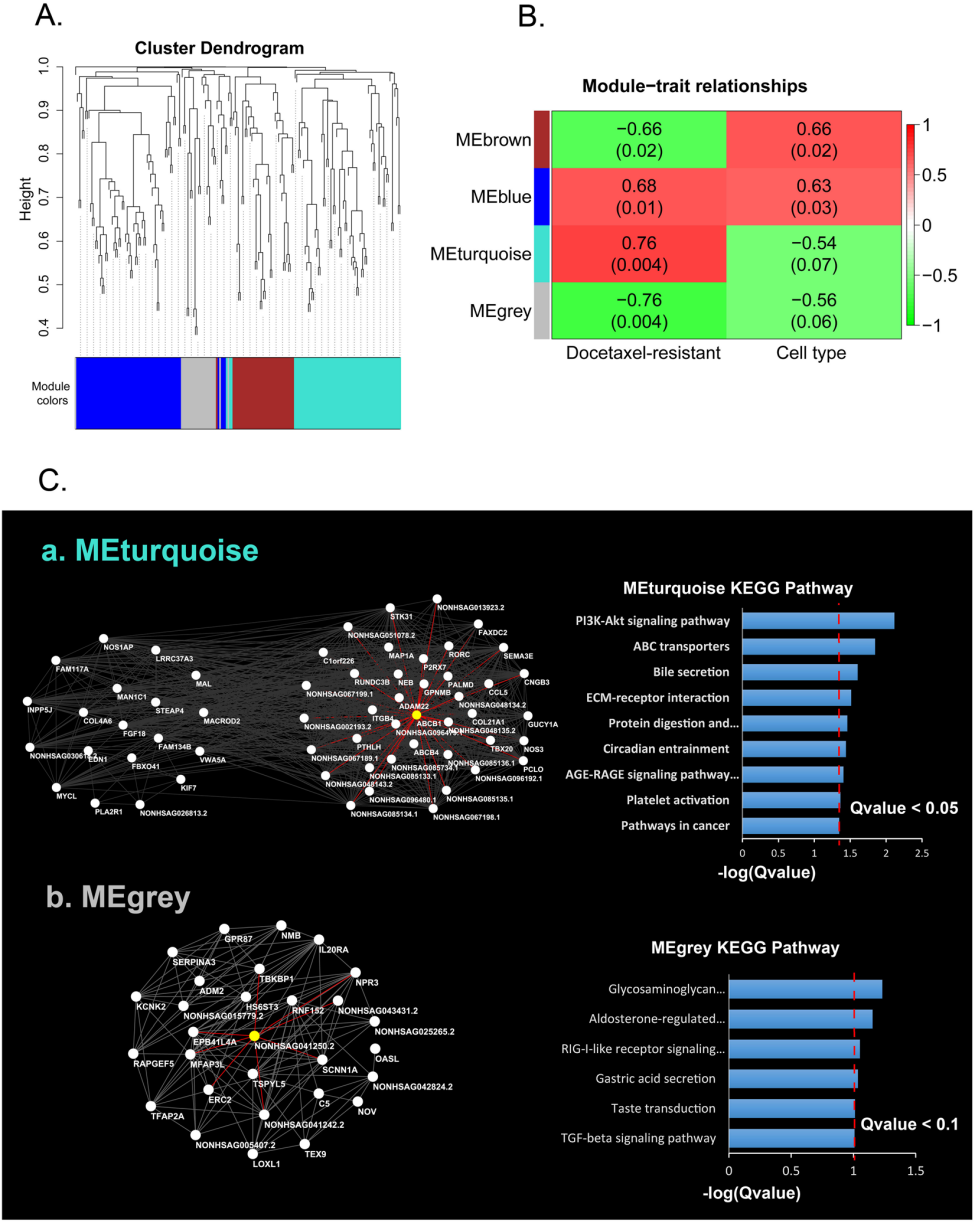
lncRNA-mRNA co-expression network construction and docetaxel-resistant module detection using the consistent SDE mRNAs and lncRNAs. (**A**) A total of 72 SDE lncRNAs and 124 SDE mRNAs were subjected to the WGCNA analysis. Using the hierarchical clustering method, 4 groups of co-expressed genes, termed ‘modules’, were identified. (**B**) The 4 modules were related to the docetaxel-resistant status. The correlation coefficients representing the module-trait relationships are indicated in the center of the modules, and the corresponding P-values are in the brackets. (**C**) The hub genes in the Turquoise and Gray modules were visualized by Cytoscape. Pathway enrichment was performed with the hub genes in the two modules. a. The connections of the 59 hub genes in the Turquoise module. The white nodes represent the hub genes, and the red lines indicate the connections to *ABCB1*. b. The connections of the 29 hub genes in the Gray module. The white nodes represent the hub genes, and the red lines indicate the connections to NONHSAG041250.2 (EPB41L4A-AS2).

All the SDE genes (72 lncRNAs and 124 mRNAs) were subjected to the WGCNA analysis. We identified 4 groups of co-expressed genes, termed ‘modules’ (Fig. 3A). Each module was labeled with a unique color underneath the cluster tree^19^.

Inside a given module, the expression profiles of the transcripts can be summarized by the ‘module eigengene’, which is the weighted average of the module gene expression profiles^19^. To identify the module associate with docetaxel-resistant status, we regressed each of the 4 module eigengenes on docetaxel-resistant status. We found that the Turquoise and Gray modules were most significantly associated with docetaxel-resistant status (Fig. 3B). Of these 2 associated modules, the Turquoise was positively correlated with docetaxel resistance, meaning that the genes in this module are predominantly up-regulated in docetaxel-resistant cells. In contrast, the Gray module was negatively correlated with a docetaxel-resistant status, meaning that most genes in this module were down-regulated in the docetaxel-resistant cells.

To identify the genes that are central and highly connected in the docetaxel-resistance-associated modules, we selected Hub genes with |GS| > 0.4 (gene significance) and |MM| > 0.8 (module membership) in the two modules and visualized these genes by Cytoscape (Fig. 3C).

In the Turquoise module, we identified 59 hub genes, including 40 protein coding genes and 19 lncRNAs (Sup Table 4). Several well-known chemotherapy-resistance-associated genes including *ABCB1* were found in the protein coding genes list. We found that 39 genes, including 22 protein coding and 17 non-coding, were linked to *ABCB1* (Table 2). Among the 22 coding genes linked to *ABCB1*, we found that many of the genes are implicated in drug or chemotherapy resistance, such as *ABCB4*, *ADAM22* (disintegrin and metalloproteinase domain-containing protein 22), *GPNMB* (glycoprotein nmb), *COL21A1* (collagen type XXI alpha 1 chain), and *ITGB4* (integrin subunit beta 4) (Table 2, marked with asterisks). Strikingly, we found a list of lncRNAs closely linked to *ABCB1* in the Turquoise module (Table 2), indicating that these lncRNAs may function as regulators of *ABCB1*.

**Table 2:** Nodes links to ABCB1 in the Turquoise module.

Node links to ABCB1	Region	Log_2_ (MCF-RES/MCF-7)	MCF padj	Log_2_ (MDA-RES/MDA-MB-231)	MDA padj
ABCB4*	chr7:87031361-87105019	4.391873563	3.0392E-81	4.798895486	1.78397E-10
ADAM22*	chr7:87563566-87832204	3.031204518	5.24574E-71	2.042465751	8.48915E-17
C1orf226	chr1:162348696-162356608	1.30617006	2.08367E-08	1.218567139	0.002493763
CCL5	chr17:34198495-34207377	4.281358914	2.28917E-14	3.425308925	0.016192984
CNGB3	chr8:87586024-87755903	2.927154653	8.53446E-07	4.3137896	4.17421E-05
COL21A1*	chr6:55921388-56112378	4.89902163	1.49237E-23	3.061434362	0.004363835
FAXDC2	chr5:154198052-154230213	1.236440283	7.16651E-08	2.057091728	0.002687024
GPNMB*	chr7:23286316-23314729	3.634939682	3.41382E-06	3.979523007	5.07143E-21
GUCY1A2	chr11:106557910-106889171	3.923885806	3.04573E-08	2.419615258	0.008199061
ITGB4*	chr17:73717516-73753899	1.264990464	3.44083E-05	1.28963119	1.4676E-10
MAP1A	chr15:43809806-43823818	1.820284794	0.005866863	2.930015235	4.37426E-12
NEB	chr2:152341853-152591001	5.071449042	1.95162E-15	1.272976565	0.074560694
NOS3	chr7:150688144-150711687	4.229375395	6.48733E-15	1.677234009	0.009459785
P2RX7	chr12:121570631-121624354	3.174388573	3.15828E-08	3.742594891	0.024211279
PALMD	chr1:100111431-100160097	3.418917876	1.04212E-12	3.476080265	0.000185888
PCLO	chr7:82383321-82792197	5.256273053	6.619E-18	1.320153124	0.000404159
PTHLH	chr12:28111017-28124916	5.660286658	2.89989E-37	1.102001097	0.022862275
RORC	chr1:151778547-151804348	2.438996823	2.19322E-10	2.525862773	0.011217021
RUNDC3B	chr7:87257729-87461613	3.889364599	2.65512E-25	2.527876138	1.00736E-12
SEMA3E	chr7:82993222-83278479	3.4028601	3.31146E-90	3.774037375	3.23296E-38
STK31	chr7:23749838-23872132	4.004572338	6.89337E-33	1.460119768	0.003048511
TBX20	chr7:35242042-35293711	3.537902823	0.000168179	3.453322279	0.025040493
NONHSAG002193.2	chr1:100111502-100152712	2.306183482	0.00000108	1.765770312	0.035443541
NONHSAG013923.2	chr13:89793303-89815434	2.858465834	4.82E-10	2.473788666	0.000298561
NONHSAG048134.2	chr7:87150777-87151726	4.661655972	4.16E-34	4.849823297	1.08E-20
NONHSAG048135.2	chr7:87229434-87342570	3.06347186	6.79E-13	3.485170506	3.16E-09
NONHSAG048143.2	chr7:87829109-87832202	3.266694083	3.73E-33	1.013242299	0.016081777
NONHSAG051078.2	chr8:115582746-115686229	3.00855	2.46E-23	1.929201108	0.008762611
NONHSAG067189.1	chr13:89594992-89616155	5.165615	7.06E-47	3.947107328	1.03E-13
NONHSAG067198.1	chr13:91322334-91371571	3.65213	2.43E-20	2.165973989	0.003256962
NONHSAG067199.1	chr13:91465148-91472109	4.027848	2.53E-24	2.187822303	0.000869085
NONHSAG085133.1	chr3:165592410-165780111	2.821382	1.38E-20	5.039315468	1.02E-23
NONHSAG085134.1	chr3:165609026-165630375	2.314128	1.26E-09	3.197328113	0.000000284
NONHSAG085135.1	chr3:165633442-165636497	2.229836	1.85E-09	3.518443205	9.92E-10
NONHSAG085136.1	chr3:165651727-165888351	1.995099	0.000138828	1.567863816	0.09203305
NONHSAG085734.1	chr3:126760621-126763555	1.264793	0.05765945	1.590391302	0.083859182
NONHSAG096192.1	chr7:23296654-23301401	2.382385	1.7E-09	3.160102464	2.15E-08
NONHSAG096479.1	chr7:87075330-87214789	7.059226	1.77E-99	7.209915426	7.23E-58
NONHSAG096480.1	chr7:87445537-87459755	2.385153266	9.27E-10	1.169846587	0.062398465

*Asterisk indicates the gene which has been implicated in chemotherapy resistant; padj refer to adjusted P value.

In the Gray module, we identified 29 hub genes, including 22 protein coding genes and 7 lncRNAs (Sup Table 5). We found that some of the protein coding genes are reported to be associated with chemotherapy sensitivity. The over-expression of *TSPYL5* (testis-specific Y-encoded-like protein 5) and *TFAP2A* (transcription factor AP-2 alpha) are reported to increase the cell sensitivity to chemotherapy in different cancers^20–23^. Moreover, in the Gray module, we also identified several lncRNAs that were consistently down-regulated in the docetaxel-resistant MCF7-RES and MDA-RES cells. Notably, an antisense lncRNA, EPB41L4A-AS2 (NONHSAG041250.2), which is reported to inhibit tumor proliferation and is associated with favorable prognoses in breast cancer^24^, was identified as a hub gene in this module.

### Location and functional analysis of the important lncRNAs and mRNAs in the resistance- associated modules

**Figure 4:**
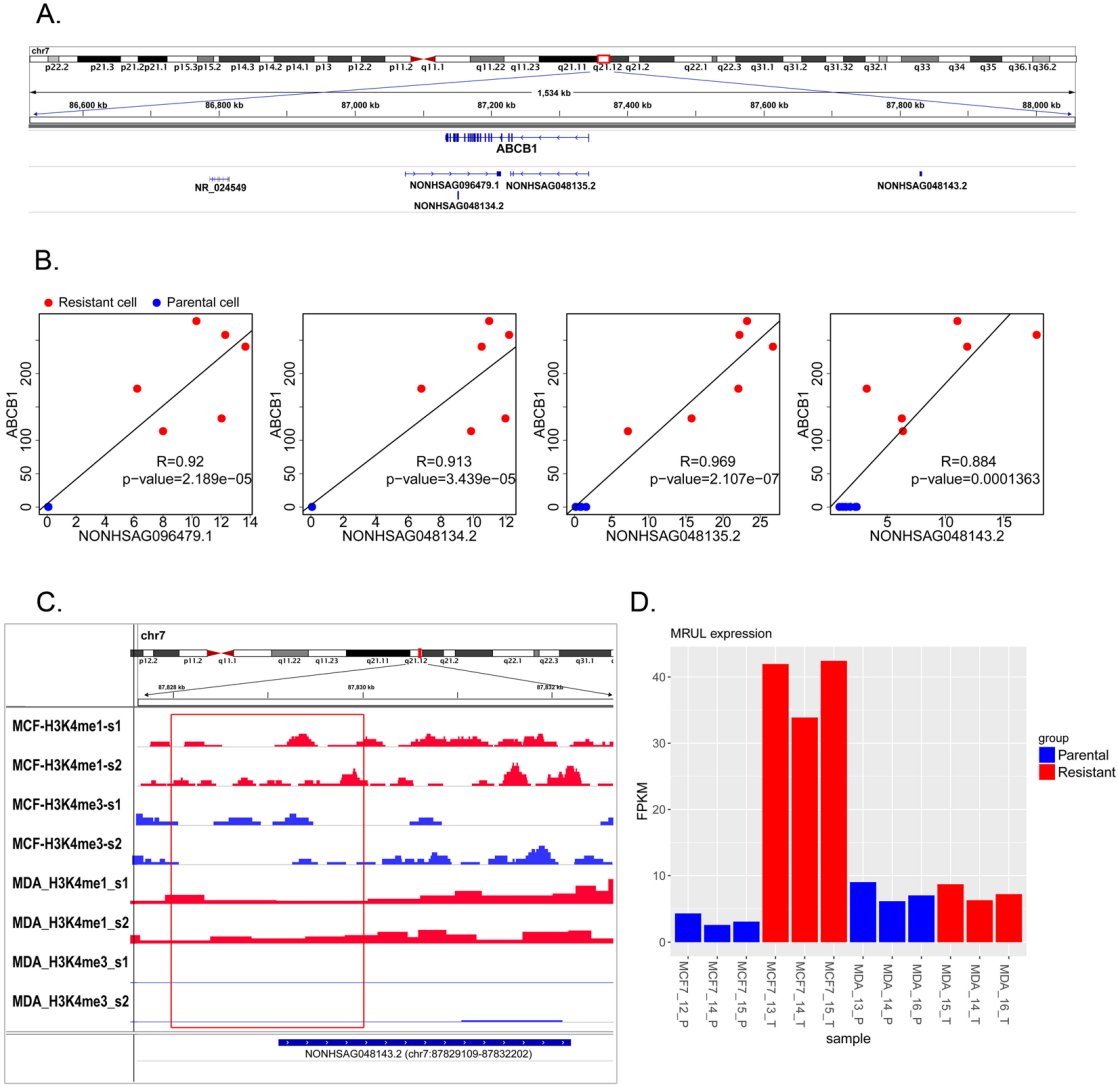
Location and functional analysis of important lncRNAs closely linked to ABCB1 mRNAs in the Turquoise module. (**A**) The location of *MRUL*, *ABCB1* and the four lncRNAs near the *ABCB1* locus. (**B**) Correlation between the expression of the 4 lncRNAs and the *ABCB1* gene. (**C**) The H3K4me1/H3K4me3 status at the TSS region (indicated by the red box) of the lncRNA NONHSAG048143.2. (**D**) The MRUL expression (FPKM) in the docetaxel-resistant cells (red bars) and parental cells (blue bars).

To uncover the relationship of the lncRNAs and the most studied MDR-related gene, *ABCB1*, we analyzed the genomic locations of the lncRNAs that were linked with *ABCB1* in the Turquoise module. Our results showed that several lncRNAs were localized at the regions overlapping or near the *ABCB1* gene locus. The locations of lncRNAs NONHSAG048134.2, NONHSAG048135.2 and NONHSAG096479.1 overlapped with *ABCB1*. NONHSAG048135.2 was transcribed in the same direction and was localized to the exons of the *ABCB1* gene, indicating that it was an exonic lncRNA, whereas NONHSAG048134.2 and NONHSAG096479.1 were located in the antisense strand to the *ABCB1* gene locus, indicating that these two lncRNAs were antisense lncRNAs for the gene *ABCB1* (Fig. 4A). Another lncRNA, NONHSAG048143.2, was located 480 kb upstream of *ABCB1* (Fig. 4A).

The correlation analysis of the expression data of these four lncRNAs and *ABCB1* mRNA revealed a strong positive correlation between the expression of these lncRNAs and the *ABCB1* gene (Fig. 4B), suggesting that there was regulatory relationship between these lncRNAs and the *ABCB1* gene.

We downloaded the ChIP-seq (chromatin immunoprecipitation sequencing) datasets for MCF-7 and MDA-MB-231 breast cancer cells from the GEO (Gene expression omnibus) and ENCODE (encyclopedia of DNA elements) databases. By calculating the H3K4me1/H3K4me3 ratios at each TSS (transcriptional start site) of the lncRNAs near *ABCB1*, we found that the lncRNA NONHSAG048143.2, upstream of *ABCB1*, was associated with an enhancer-like chromatin state (H3K4me1/H3K4me3 ratio > 1.2) in both the MCF-7 and MDA-MB-231 cells (Fig. 4C). This indicated that NONHSAG048143.2 might function as an eRNA (enhancer lncRNA)^25^ in the regulation of the *ABCB1* gene.

We also identified a previously reported lncRNA, NR_024549 (termed MRUL)^10^, which located 400 kb downstream of *ABCB1*. This lncRNA was found to be down-regulated in MCF7-RES cells (Fig. 4D).

**Figure 5:**
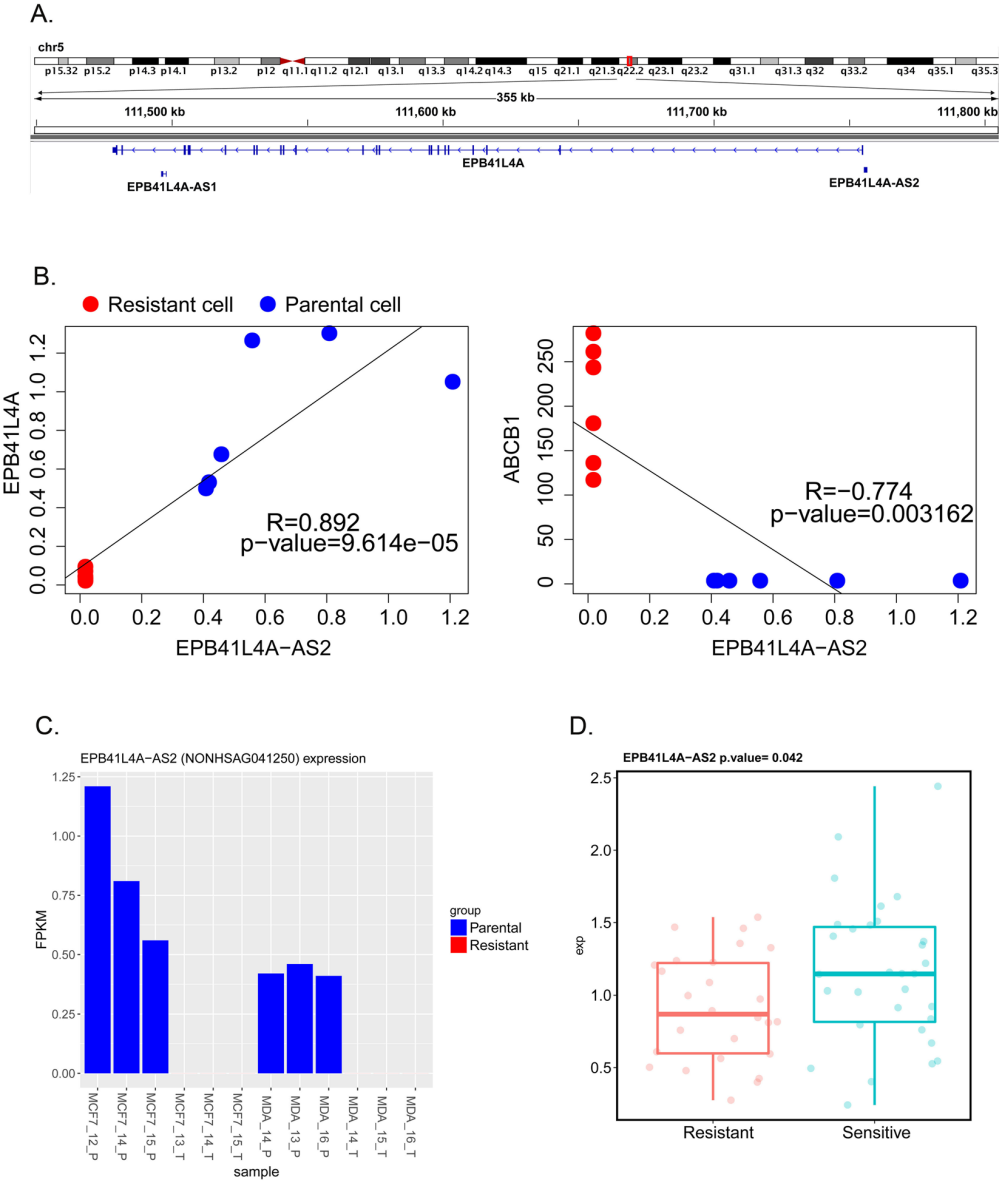
Location and functional analysis of the lncRNA EPB41L4A-AS2. (**A**) The location of the *EPB41L4A-AS2* and *EPB41L4A* genes. (**B**) Correlation between the expression of *EPB41L4A-AS2* and *EPB41L4A* and *ABCB1*. (**C**) The expression of *EPB41L4A-AS2* (FPKM) in the docetaxel-resistant cells (red bars) and parental cells (blue bars). (**D**) The expression of *EPB41L4A-AS2* in clinical breast cancers tissues (GSE21997) before neoadjuvant chemotherapy (doxorubicin and docetaxel).

The other lncRNA, EPB41L4A-AS2, which had been reported to be a potential tumor suppressor and prognostic biomarker was located head to head with the *EPB41L4A* gene in the antisense orientation (Fig. 5A). The correlation analysis on the expression data of this lncRNA and EPB41L4A and *ABCB1* mRNAs revealed strong positive correlation between the expression of EPB41L4A-AS2 and EPB41L4A (Pearson correlation test, R = 0.892, P = 9.61 × 10^−5^, Fig. 5B) but strong negative correlation between the expression of EPB41L4A-AS2 and *ABCB1* genes (Pearson correlation test, R = 0.774, P = 0.003, Fig. 5B).

To validate the relationship between EPB41L4A-AS2 expression and chemotherapy resistance, we downloaded the primary microarray data GSE21997, which profile the RNA expression in primary breast cancers before neoadjuvant chemotherapy (doxorubicin and docetaxel)^26^, from the GEO repository database. And the residual breast cancer burden (RCB) index was adopted to classified the breast cancer patients as previous report^27^. Patients of RCB-III class were considered to be therapeutic resistant. By analyzing the microarray data, we found that the expression of EPB41L4A-AS2 was significantly lower (Student’s t-test, P = 0.042, Fig. 5D) in the resistant group than the non-resistant group.

### Real-time qPCR validation of important SDE lncRNAs and mRNAs

**Figure 6:**
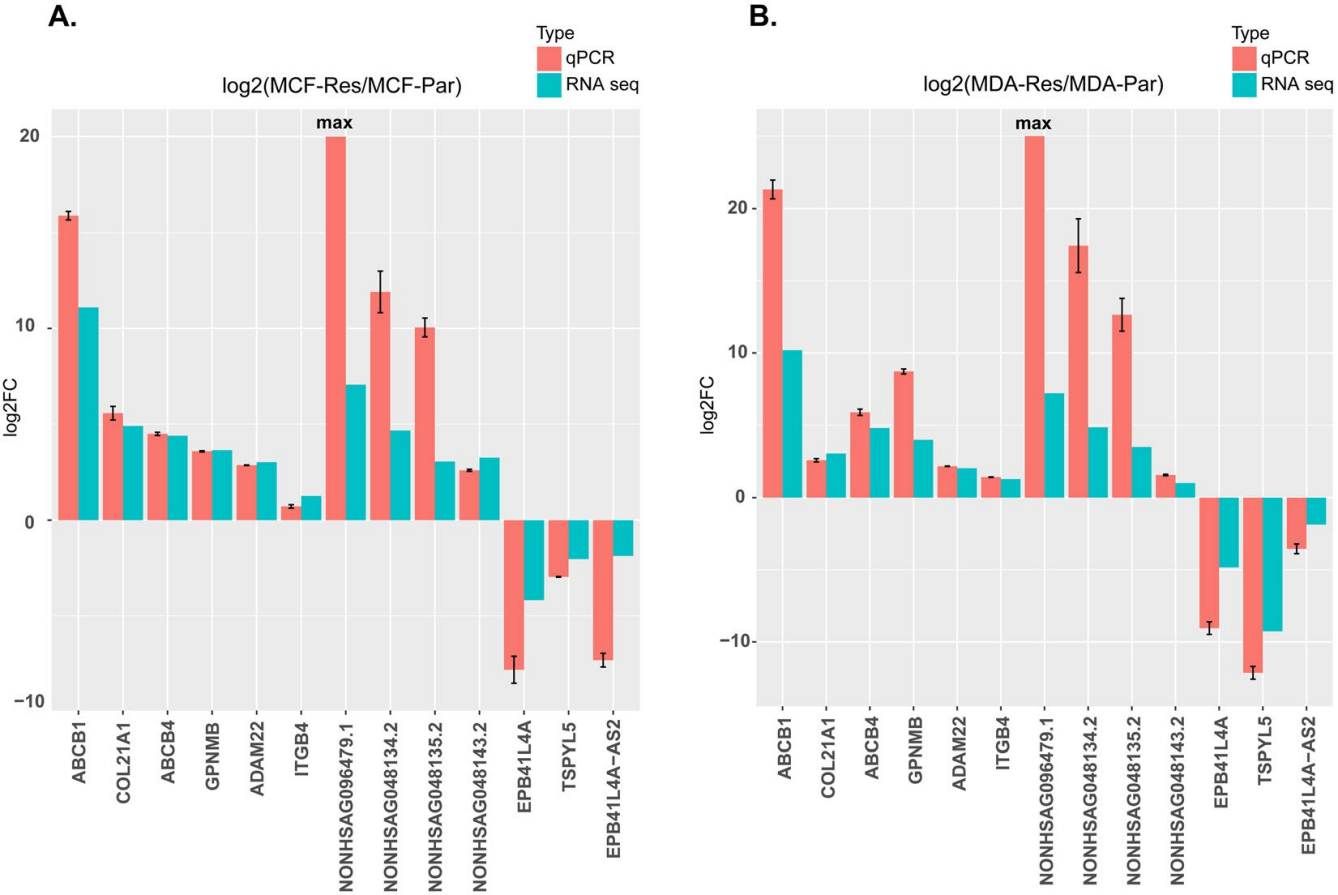
Real-time qPCR validation of important SDE lncRNAs and mRNAs. Validation of the important SDE lncRNAs and mRNAs in the MCF cells (**A**) and MDA cells (**B**). The heights of the red columns represent the logarithmically transformed mean fold-changes between the resistant and parental cells detected by QPCR. Data from three duplications are presented as mean ± standard error. The blue columns represent the logarithmically transformed fold-changes between the resistant and parental groups detected by RNA-seq. ‘Max’ represents an infinitely large number due to the absence of NONHSAG096479.1 in the parental cells.

The qPCR results showed that the expression of the mRNAs, including ABCB1, ABCB4, ADAM22, COL21A1, GPNMB, and ITGB4 and the expressions of the lncRNAs, including NONHSAG096479.1, NONHSAG048134.2, NONHSAG048135.2, and NONHSAG048143.2 were up-regulated in docetaxel resistant MCF7-RES and MDA-RES cell lines compared to their parental cells lines. On the contrary, the expressions of the mRNAs EPB41L4A and TSPYL5 and the expressions of the lncRNAs EPB41L4A-AS2 were down-regulated in docetaxel resistant MCF7-RES and MDA-RES cell lines compared to their parental cells lines (Fig. 6). The deregulated genes were identically regulated in both the MCF (Fig. 6A) and MDA (Fig. 6B) groups. Importantly, the qPCR analyses validated the results obtained by RNA-seq data.

## Discussion

Taxanes are a class of anti-microtubule agents that have been demonstrated to be more efficient than anthracycline-based regimens and are listed as the first-line regimens for breast cancer^28,29^. However, patients receiving taxane treatment usually develop resistance to taxanes, thus resulting in recurrence and metastasis^30^.

To uncover the mechanisms underlying taxane resistance and find novel potential predictive biomarkers for clinical use, many studies have been carried out in both clinical cancer samples and resistant cell model systems^4,31–38^. Through this research, a number of genes were identified to be associated with taxane resistance, and the differential expression of the *ABCB1* gene was extensively investigated and identified as one of the most credible biomarkers in chemotherapy-resistant cancers^3,4,33,34,36,37^. However, these previous studies were mainly based on microarray hybridization technologies, and therefore, limited concordant differentially expressed genes are shared between the different studies^3,4,33,34,36,37^, and the common denominator associated with taxane resistance remains largely unknown. Moreover, the underlying mechanism of taxane resistance in breast cancer and the regulators of taxane-resistant genes still needs to be uncovered.

lncRNAs regulate gene expression at the transcriptional and epigenetic levels^39^. Recent studies have shown that lncRNAs are also implicated in tumor chemotherapy resistance^9–13^. However, the contribution of lncRNAs to docetaxel resistance in breast cancer is still unclear.

Here, we used RNA-Seq technology (Illumina platform), which does not rely on pre-designed complement probes and shows a higher sensitivity in the detection of genes with very low expression and a higher accuracy in the detection of extremely abundant genes compared with microarray technology^40^, to gain a more comprehensive and global profile of mRNAs and lncRNAs in docetaxel-resistant breast cancer cells (MCF7-RES and MDA- RES).

We detected an average of 26,329 lncRNAs and 13,010 mRNAs per sample. Importantly, the lncRNAs and mRNAs showed a high consistency between different passages from the same cell subline (Sup Figs 1A, 4A), which was in concordance with the reports on the high reproductivity of Illumina RNA-Seq^40^ and demonstrated the reliability of these data.

We identified 1630 and 957 SDE coding genes in the MDA-RES and MCF7-RES groups compared to their parental cells, respectively. In comparison to the previous microarray analysis research in the same cell lines^4^, we identified more consistently up-regulated or down-regulated genes shared by the MCF7-RES and MDA-RES cells (124 vs 16), which are the potential common denominators associated with taxane resistance. The KEGG pathway analyses were performed on the SDE genes in each cell line, which identified the common pathways shared by the two cell lines and found that the most enriched pathways were different between the two cell lines (Sup Figs 2, 3). The 124 consistent SDE genes were also significantly enriched in 14 signaling pathways (Q-value < 0.05). The most significantly enriched pathway was the cGMP-PKG signaling pathway. However, the ABC transporters, the PI3K-Akt signaling pathway, and the AGE-RAGE signaling pathway in diabetic complications signaling pathway were also significantly enriched. Cyclic GMP (cGMP) is the intracellular second messenger that mediates the action of nitric oxide (NO) and natriuretic peptides (NPs), regulating a broad array of physiological processes. The role of this pathway in chemotherapy is unclear. However, it is implicated in the regulation of apoptosis in cancer cells^41^ and has been reported to mediate the inhibition of Pgp efflux function by YC-1^42^. The PI3K-Akt signaling pathway was the most significantly enriched pathway in the MDA-MB-231 cells (Sup Fig. 3) and was also significantly enriched in the MCF-7 cells. Although it has not been reported in the previous taxane-resistance studies in breast cancer^3,4,33,34,36,37^, we found that it was linked to docetaxel resistance in prostate cancer^43,44^. These results indicated that in addition to the classical pathways, such as the ABC transporters and ECM-receptor interaction pathways, other signaling pathways may also play important roles in taxane resistance in breast cancer.

In addition to the mRNA analysis, the more novel and important work here was the profiling of the lncRNAs in the parental and docetaxel-resistant breast cancer cells. We identified 72 consistently SDE lncRNAs (50 up-regulated and 22 down-regulated) in both of the docetaxel-resistant MCF-7 and MDA-MB-231 cell lines. By performing complete-linkage clustering with these consistently SDE lncRNAs, the 12 samples (3 samples for each of the 4 cell lines) perfectly clustered into two groups: the parental and resistant groups (Fig. 2D). A comparison of the clustering results with a similar number of SDE mRNAs (Fig. 1C) suggested that lncRNAs might be more specific biomarkers for distinguishing docetaxel-resistant cells from the non-resistant cells than mRNAs. Furthermore, this highlighted the potential application of lncRNAs as predictive biomarkers and for the prediction of response to chemotherapy.

lncRNAs have been described to exert their biological functions by regulating the generation and expression of target mRNAs^8^. Co-expression network analyses have been widely used to predict the target mRNAs of lncRNAs by detecting their similar expression patterns^15,16^, and thus, the WGCNA R software package was used in our study to decipher the relationship between the SDE mRNAs and the SDE lncRNAs. By using a hierarchical clustering method, we identified 4 groups of co-expressed lncRNAs and mRNAs, termed ‘modules’. Among these, the modules “Turquoise” and “Gray” were most significantly associated with a docetaxel-resistant status.

In the Turquoise module, which was positively correlated with docetaxel resistance, *ABCB1* was identified as one of the central and highly connected genes. We found that 39 genes, including 22 coding and 17 non-coding, were linked to *ABCB1* in this module. In the 22 coding genes linked to *ABCB1*, we found that many of the genes were implicated in drug or chemotherapy resistance. For instance, *ABCB4*, another ATP-binding cassette gene, which was reported to be involved in taxane resistance^7^, also clustered in this module. *ADAM22* was also closely linked to *ABCB1*. This gene has been reported to function as an estrogen receptor-independent mediator of endocrine-resistant breast cancer^45^. However, it has not previously been linked to docetaxel resistance. Similarly, the gene *GPNMB* was also linked to *ABCB1* in the Turquoise module and was up-regulated in all the docetaxel-resistant cells. *GPNMB* has been shown to induce aggressive cellular phenotypes in breast cancer and has been identified as a potential therapeutic target for patients with basal-like breast cancer (BLBC) and TNBC^46,47^. The concordant expression of *ABCB1* and other potential drug-resistant genes in docetaxel-resistant breast cancer cells indicates that besides *ABCB1*, there are also other genes that may play an important role in the generation of docetaxel resistance, and these genes may contribute to docetaxel resistance in a cooperative way with *ABCB1*. Pathway enrichment with the genes in this module highlighted the important roles of the PI3K-Akt and ABC transporter signaling pathways.

Moreover, a striking finding here is that many lncRNAs were also co-expressed with *ABCB1* and other potential drug-resistant genes in this module. We identified a list of lncRNAs closely linked to *ABCB1* in the Turquoise module (Table 2), indicating that some of these lncRNAs may function as regulators of *ABCB1* By analyzing the genomic location of the lncRNAs linked with *ABCB1*, we found that several lncRNAs were located at the regions overlapping or near the *ABCB1* gene locus. The lncRNA NONHSAG048135.2 was identified as an exonic lncRNA of *ABCB1*, whereas NONHSAG048134.2 (termed ABCB1-AS1) and NONHSAG096479.1 (termed ABCB1-AS2) were identified as antisense lncRNAs for *ABCB1* Another lncRNA, NONHSAG048143.2, was identified as an intergenic lincRNA located 480 kb upstream of *ABCB1*.

lncRNAs are implicated in a wide-spectrum of biological processes at different molecular levels, including epigenetic modifications, transcription, and post-transcriptional processing^48^, and the functions of lncRNAs appears to always to be associated with the location of the lncRNA and the nearby mRNA^49,50^. Antisense lncRNAs have been shown to bind to the complementary pre-mRNAs, regulate RNA editing and splicing and, finally, affect the expression levels of mRNAs and proteins^51,52^. For instance, lncRNA ZEB2 NAT (zinc finger e-box binding homeobox 2 natural antisense transcript) was reported to bind to the ZEB2 pre-mRNA and obstruct the splicing of the Zeb2 5’-UTR, thus up-regulating the expression of the Zeb2 protein^52^. Antisense lncRNAs have also been shown to increase the stability of mRNAs by forming a lncRNA-mRNA duplex and, thereby, up-regulate gene expression^53,54^. Although, the exact functions of the two novel antisense lncRNAs ABCB1-AS1 and ABCB1-AS2 are still unclear, and further research needs to be performed, our correlation analysis revealed a strong positive correlation between the expressions of these antisense lncRNAs and ABCB1 mRNA (Fig. 4B) and raised the possibility that these lncRNAs may up-regulate the expression of the *ABCB1* gene via RNA splicing or stabilization mechanisms.

In addition to the lncRNAs located within the locus of the mRNAs, accumulating evidence suggests that lncRNAs located in the regulatory regions are also critical to the regulation of gene expression^39,55^. Many lncRNA transcripts from enhancer regions have been shown to facilitate the transcription of protein-coding genes^56^. For instance, CCAT1-L, which is transcribed from a super-enhancer region 500 kb upstream of the *MYC* (Proto-Oncogene C-Myc) gene, promotes chromatin interactions between *MYC* and its upstream regulatory elements and, thereby, up-regulates *MYC* transcription^57^. Recently, a study of lncRNAs in doxorubicin- and vincristine-resistant gastric cancer cells also identified an lncRNA, MRUL, which is located 400 kb downstream of *ABCB1* and promotes the expression of *ABCB1*^10^. To our knowledge, this is the only lncRNA reported to promote *ABCB1* expression in chemotherapy-resistant cancer cells. Since this lncRNA has not been recorded in the NONCODE 2016 database, we analyzed the differential expression of the transcripts of MRUL between the parental MCF-7 and MDA-MB-231 cells and their docetaxel-resistant sublines. We found that MRUL was dramatically up-regulated in the docetaxel-resistant MCF-7 subline compared to the parental MCF-7 cells. However, this up-regulation was not apparent in the docetaxel-resistant MDA-MB-231 subline (Fig. 4D). This finding suggested that MRUL may also contribute to docetaxel resistance in ER (estrogen receptor) positive breast cancer but maybe not in the TNBC type.

Most intriguingly, in our study, we also identified the lncRNA NONHSAG048143.2 located 480 kb upstream of *ABCB1*. The correlation analysis revealed a strong positive correlation between the expression of this lncRNA and ABCB1 mRNA (Fig. 4B), suggesting that this lncRNA may be another enhancer lncRNA for *ABCB1*. The histone H3K4me1/H3K4me3 ratio has been used to characterize enhancer elements across the genome^25,58,59^. Therefore, we downloaded the ChIP-seq datasets of the MCF-7 and MDA-MB-231 breast cancer cells to calculate the H3K4me1/H3K4me3 ratios at the TSS of the lncRNA NONHSAG048143.2. Our result revealed that lncRNA NONHSAG048143.2 was located in a region presenting an enhancer-like chromatin state (H3K4me1/H3K4me3 ratio > 1.2) in both the MCF-7 and MDA-MB-231 cells (Fig. 4C). This result strongly suggested that this lncRNA might function as an enhancer lncRNA in the up-regulation of the *ABCB1* gene in docetaxel-resistant breast cancer cells.

In the Gray module, which was negatively correlated with the docetaxel-resistant status, we found that the gene *TSPYL5* was dramatically and consistently down-regulated in the docetaxel-resistant MCF-7 and MDA-MB-231 cells. This gene has been involved in the modulation of cell growth and the cellular response to gamma radiation probably via the regulation of the Akt signaling pathway^60^. The overexpression of *TSPYL5* has also been linked to chemotherapy sensitivity in both lung and prostate cancer cells^20,61^. The dramatic down-regulation of *TSPYL5* in the docetaxel-resistant breast cancer cells may be involved in the loss of docetaxel sensitivity. Interestingly, we found that the lncRNA EPB41L4A-AS2 was expressed in all the parental breast cancer cell lines but was absent in all the docetaxel-resistant descendants (Fig. 5C). According to a recent study, this lncRNA may inhibit tumor cell proliferation and is associated with favorable prognoses in breast cancer^24^. However, the role of EPB41L4A-AS2 in chemotherapy-resistant cancer has not been deciphered yet. Here, we report, for the first time, the depletion of EPB41L4A-AS2 in docetaxel-resistant breast cancer cells. Our correlation analysis also showed that the decreased level of EPB41L4A-AS2 in the docetaxel-resistant breast cancer cells was significantly associated with an increased level of ABCB1 mRNA (Fig. 5B). These results strongly indicate that the depletion of EPB41L4A-AS2 contributes to the docetaxel-resistant phenotype in breast cancer cells. In support of this notion, our analysis in clinical breast cancers tissues (GSE21997) before neoadjuvant chemotherapy (doxorubicin or docetaxel) also showed that the expression of EPB41L4A-AS2 was significantly lower in the patients resistant to neo-adjuvant chemotherapy than in non-resistant patients (Fig. 5D).

In conclusion, we performed a comprehensive Illumina-based RNA sequencing and analysis on two docetaxel-resistant breast cancer cell lines and their parental breast cancer cell lines. In addition to the most studied docetaxel-resistant gene, *ABCB1*, we also identified other SDE protein-coding genes and pathways, which might contribute to the generation of docetaxel resistance. These findings improved our understanding of the mechanism underlying docetaxel resistance in breast cancer and suggest that a number of lncRNAs are promising candidates as predictive biomarkers for the docetaxel response. To our knowledge, this is the first study to report on a global profile of mRNAs and lncRNAs in docetaxel-resistant breast cancer. We identified groups of lncRNAs, which were consistently up-regulated or down-regulated in both the docetaxel-resistant MCF-7 and MDA-MB-231 cell lines and constructed a co-expression network to decipher the potential regulatory relationship between the SDE lncRNAs and mRNAs. Our results highlighted four overexpressed lncRNAs that overlapped or were near the *ABCB1* locus, which might up-regulate the expression of *ABCB1* via different mechanisms. We also identified lncRNA EPB41L4A-AS2 as a potential biomarker for docetaxel sensitivity in clinical breast cancer samples. Although further basic biological and clinical research still needs to be performed to explore the exact biological function of these newly identified lncRNAs, our study provided potential biomarkers to predict the response to docetaxel in breast cancer patients and might help improve the strategy for the treatment of advanced breast cancer.

## Methods

### Cell culture and treatment

The two human breast cancer cell lines MDA-MB-231 and MCF-7 and their docetaxel-resistant sublines MDA-RES and MCF7-RES were obtained from Hansen *et al*.’s study^4^.

The MCF-7 cells were cultured in DMEM (Gibco, Thermo Fisher Scientific, USA) plus 1% non-essential amino acids (Gibco, Thermo Fisher Scientific, USA) and 5% fetal calf serum (FCS) (Gibco, Thermo Fisher Scientific, USA). The MDA-MB-231 cells were cultured in DMEM plus 10% FCS. The culture medium for the MDA-RES and MCF7-RES cells was the same as their parental cells except for the supplementation of 65 or 150 nM of docetaxel (Sanofi Aventis, France), respectively. All the cell lines were grown in 5% carbon dioxide at 37 °C.

### Total RNA purification and rRNA depletion transcriptome Sequencing

For the Illumina-based RNA-Seq, the total RNA from 3 passages of MCF-7, MCF7-RES, MDA-MB-231 and MDA-RES, was purified with RNAiso Plus Kit (TAKARA, Japan). After the RNA purification and DNase I digestion, rRNAs were removed from the total RNA with RiboMinus Eukaryote Kit (Qiagen, USA). The remained RNAs were fragmented using Ambion Fragmentation Solution (Thermo Fisher Scientific, USA). Then the mRNA fragments were used to synthesize cDNAs, followed by end repairing and adenine connection. Sequencing adaptors were ligated the fragments. Suitable fragments were selected according to the agarose gel electrophoresis results for PCR amplification. The StepOnePlus System (Applied Biosystems, Thermo Fisher Scientific, USA) and the Agilent 2100 Bioanaylzer (Agilent Technologies, USA) were used to quantify and qualify the sample libraries in the QC steps. Finally, all the libraries were sequenced by the HiSeq. 2000 sequencer (Illumina, USA).

### lncRNA and mRNA expression profiling

Firstly, adaptor sequences were removed from the raw reads. And low quality reads, which contained more than 50% of low quality bases (base quality < 10) and 5% of undefined nucleotides [N] on the read, were also removed. To avoid the reads mapped to the remaining rRNAs disturbing the next analysis, we aligned the reads to the human ribosomal RNA sequences (rRNA) and filtered out the perfect mapping reads. After the above two-step filtering, the remaining reads were considered clean reads and were used in next expression profile. In the mRNA analysis, the clean reads were mapped to the human reference genome (hg19) by TopHat2^62^. Then, the genes annotated in RefSeq were quantified by Cufflinks tools^63^. For lncRNA, the clean reads were mapped to the noncoding RNA transcriptome (NONCODE 2016^64^) by STAR^65^ and quantified by RSEM^66^. The parameters used in the analysis tools referred to the ENCODE RNA-seq data processing pipelines (https://www.encodeproject.org/pipelines/). Finally, we obtained all the mRNA and lncRNA expression tables represented in both the FPKM and read-count format.

### Identification of significantly differentially expressed lncRNAs and mRNAs

The differentially expressed lncRNAs and mRNAs between the parental and docetaxel-resistant cells groups were calculated by DESeq.^67^ in the two cell lines, respectively. To ensure that the denominator was not zero when performing the division operation, we added 1 to every element in the read-count matrix. The P-value was calculated based on a negative binomial distribution model, and adjusted by Benjamini-Hochberg mothed. The criteria of |Log2FC| > 1, FDR < 0.1 was used to select the significantly differentially expressed lncRNAs and mRNAs.

### GO and KEGG pathway enrichment

Functional annotation enrichment analyses for GO and the KEGG pathway were performed using the KOBAS server^68^. P-value is calculated by hypergeometric test. The Q-value is the P-value corrected by Benjamini-Hochberg mothed. The GO terms and pathways with a Q value less than 0.05 were considered significantly enriched.

### Co-expression network analysis

The WGCNA R package^18^ was used to construct the co-expression network between the significantly differentially expressed lncRNAs and mRNAs.

By hierarchical clustering of the expression data, several modules, which are clusters of highly interconnected genes, were identified in the co-expression network. Then, we related the drug-resistant feature to the module eigengene to identify the drug-resistant modules (Pearson correlation test, P-value < 0.05). In the drug-resistant modules, hub genes were selected by |MM| > 0.8 and |GS| > 0.4, and they were highly connected with other genes. The connections among the hub genes were visualized as an unsigned network by Cytoscape^69^.

### Classification of lncRNAs near ABCB1 with the downloaded ChIP-seq (H3K4me1/H3K4me3) data

We downloaded the ChIP-seq datasets for the MCF-7 and MDA-MB-231 breast cancer cells from the GEO and ENCODE website. The NCBI (National Center for Biotechnology Information) accession number for the MDA-MB-231 cell ChIPseq data is GSE49651. The ENCODE accessions for the MCF-7 cell datasets are ENCSR493NBY and ENCSR985MIB. Then, we calculated the H3K4me1/H3K4me3 ratio at each TSS (mean over interval, 1 kb up and down TSS).

### Validation of the SDE mRNAs and lncRNAs

The PCR primers for the eight selected mRNAs (including ABCB1, ABCB4, ADAM22, COL21A1, GPNMB, ITGB4, and EPB41L4A) and the five lncRNAs (including EPB41L4A-AS2, NONHSAG096479.1, NONHSAG048134.2, NONHSAG048135.2, and NONHSAG048143.2) were designed using Primer 5.0 (Sup Table 6). The PCR primers for GAPDH was also designed to serve as the endogenous control. The length of the PCR products ranged from 100 bp to 400 bp (Sup Table 6). Total RNAs from each cell line was extracted using RNAiso Plus (TAKARA, Japan), and examined Qubit fluorometer (Invitrogen, Thermo Fisher Scientific, USA). QPCR was performed in triplicate on an StepOnePlus instrument (Applied Biosystems, Thermo Fisher Scientific, USA) using the SYBR Premix Ex Taq II (TAKARA, Japan) reagent. ROX dye II was applied for baseline correction. The relative expressions of the mRNA and lncRNAs were calculated with the 2^−ΔΔ^Ct method.

## Electronic supplementary material

Supplementary information

Dataset 1

Dataset 2

Dataset 3

Dataset 4

Dataset 5

Dataset 6

Dataset 7

Dataset 8

Dataset 9

Dataset 10
