# Supplementary material for: lncRNA profile study reveals the mRNAs and lncRNAs associated with docetaxel resistance in breast cancer cells
Source: Sci Rep. 2018 Dec 19;8:17970. doi: 10.1038/s41598-018-36231-4 (PMC6299474; doi:10.1038/s41598-018-36231-4)
Supplement: Supplementary file 1 — Supplementary information [file 41598_2018_36231_MOESM1_ESM.docx]

**lncRNA profile study reveals the mRNAs and lncRNAs associated with docetaxel resistance in breast cancer cells**

Peide Huang^1,3,4,5^, Fengyu Li^3,5^, Lin Li^3,5^, Yuling You^3^, Shizhi Luo^3^, Zhensheng Dong^3^, Qiang Gao^3^, Song Wu^2^*, Nils Brünner^1^*, Jan Stenvang^1^*

^1^ Section of Pharmacotherapy, Department of Drug Design and Pharmacology, Faculty of Health and Medical Sciences, University of Copenhagen, 2200 Copenhagen N, Denmark.

^2^ The Affiliated Luohu Hospital of Shenzhen University, Shenzhen Luohu Hospital Group, Shenzhen, China.

^3^ BGI Genomics, BGI­Shenzhen, Shenzhen, 518083 China.

^4^ The First Affiliated Hospital of Shenzhen University, Shenzhen, China.

^5^ These authors contributed equally to this work.

* Correspondence should be addressed to Song Wu, email: doctor_wusong@126.com, Nils Brünner, email: nbr@sund.ku.dk, and Jan Stenvang, email: stenvang@sund.ku.dk

**Supplementary Tables**

**Sup Table 1. The top ten significantly up or down-regulated genes in MDA-RES and MCF-RES cells.**

**Sup Table 2.** **Consistently up-regulated or down-regulated mRNAs for MDA-RES and MCF-RES cells.**

**Sup Table 3. Consistently up-regulated or down-regulated lncRNAs for MDA-RES and MCF-RES cells.**

**Sup Table 4. 59 hub genes in the Turquoise module.**

Node in the table represent a mRNA or a lncRNA in the module; Edge represent the co-expressed link between two nodes; MM (module membership) is defined as the correlation of the gene expression and the module eigengene (the first principal component of a given module); GS (gene significance) is defined as the correlation between gene and the trait.

**Sup Table 5. 29 hub genes in the Grey module.**

Node in the table represent a mRNA or a lncRNA in the module; Edge represent the co-expressed link between two nodes; MM is defined as the correlation of the gene expression and the module eigengene (the first principal component of a given module); GS is defined as the correlation between gene and the trait.

**Sup Table 6. QPCR primers and the product size**

**Supplementary Figures**

**Sup Fig. 1.** **Consistency of mRNA expression between different samples.**

A. Three-way Venn diagram showing the intersection of mRNAs from different passages of the same cell line (For the sample names, P represents the parental cells, T represents the docetaxel-resistant cells, number represents the passage). B. Principal component analysis (PCA) of mRNA expression data of docetaxel-resistant and parental cell lines. The principal component 1 (PC1) describes the global mRNA expression variance between MCF7 and MDA cell lines, and PC2 describes the global mRNA expression variance between the parental and resistance cells. The red dots indicate resistant cells, and the black dots indicate parental cells.

**Sup Fig. 2. KEGG pathway analysis of** **significantly differentially expressed genes in MCF-RES cells.**

Scatter plot for KEGG enrichment of the SDE mRNA in the MCF7 cells. RichFactor is the ratio of the consistent SDE genes annotated in a pathway to all genes in this pathway. The Q value is a corrected p-value, and the pathways with a Q value < 0.05 are showed in the figure.

**Sup Fig. 3. KEGG pathway analysis of significantly differentially expressed genes in MDA-RES cells.**

Scatter plot for KEGG enrichment of the SDE mRNA in the MDA cells. RichFactor is the ratio of the consistent SDE genes annotated in a pathway to all genes in this pathway. A Q value is a corrected p-value, and the pathways with a Q value < 0.05 were showed in the figure.

**Sup Fig. 4. Consistency of lncRNA expression between different samples.**

A. Three-way Venn diagram showing the intersection of lncRNAs from different passages of the same cell line. B. PCA of lncRNA expression data of docetaxel-resistant and parental cell lines. PC1 describes the global lncRNA expression variance between MCF7 and MDA cell lines, and PC2 describes the global lncRNA expression variance between the parental and resistance cells. The red dots indicate resistant cells, and the black dots indicate parental cells.
