# Supplementary figures and images for: lncRNA profile study reveals the mRNAs and lncRNAs associated with docetaxel resistance in breast cancer cells
Source: Sci Rep. 2018 Dec 19;8:17970. doi: 10.1038/s41598-018-36231-4 (PMC6299474; doi:10.1038/s41598-018-36231-4)

Sup Fig.1

A.

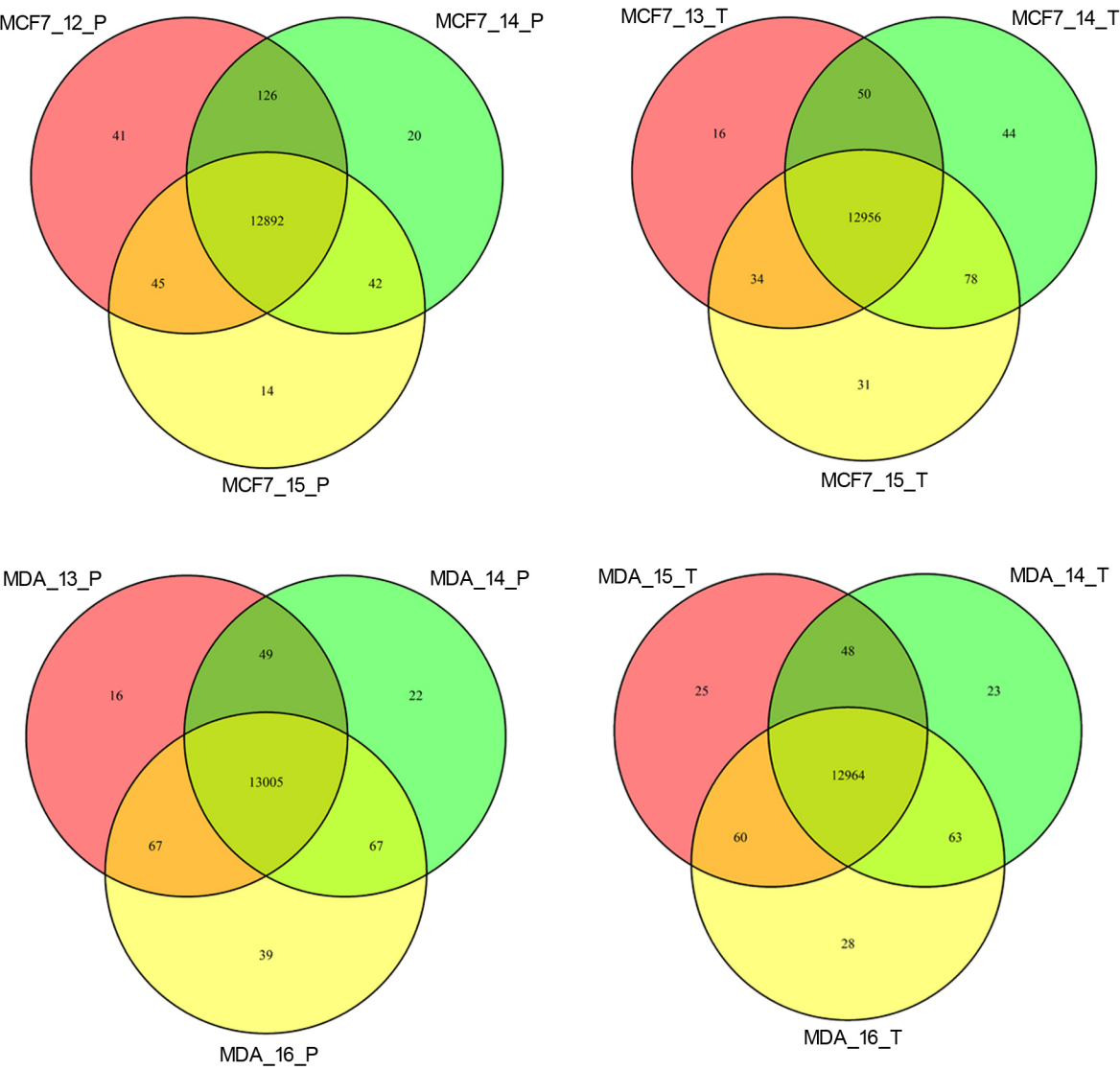

B.

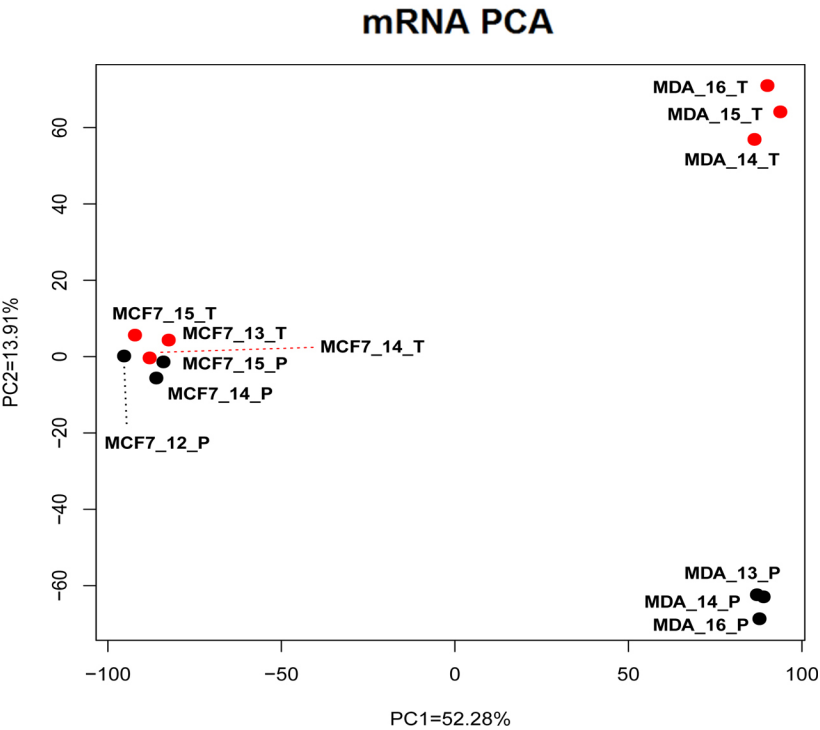

Supplement: Supplementary file 2 — Dataset 1 [file 41598_2018_36231_MOESM2_ESM.pdf]

Sup Fig.2

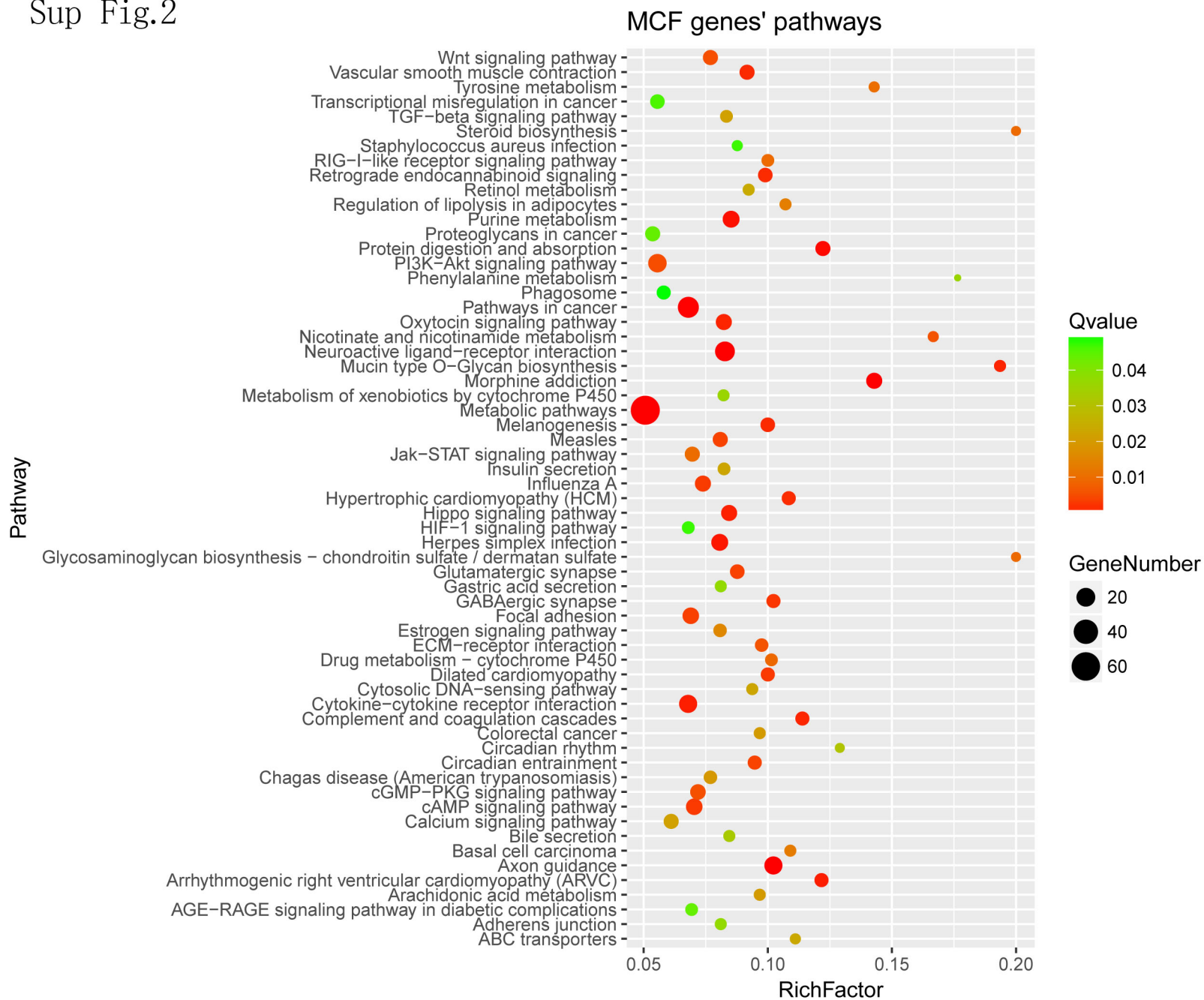

Supplement: Supplementary file 3 — Dataset 2 [file 41598_2018_36231_MOESM3_ESM.pdf]

Sup Fig.3

MDA genes' pathways

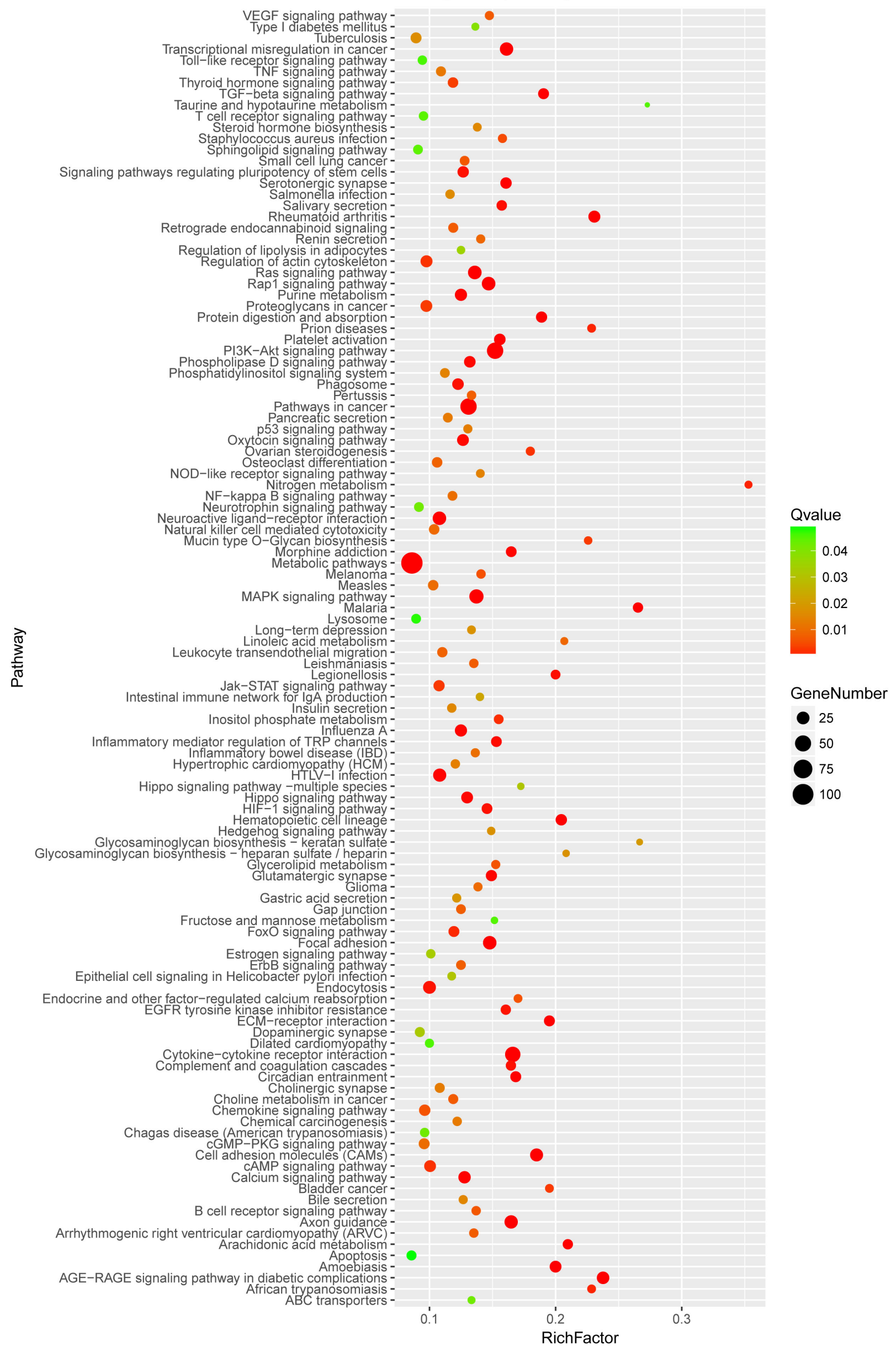

Supplement: Supplementary file 4 — Dataset 3 [file 41598_2018_36231_MOESM4_ESM.pdf]

Sup Fig.4

A.

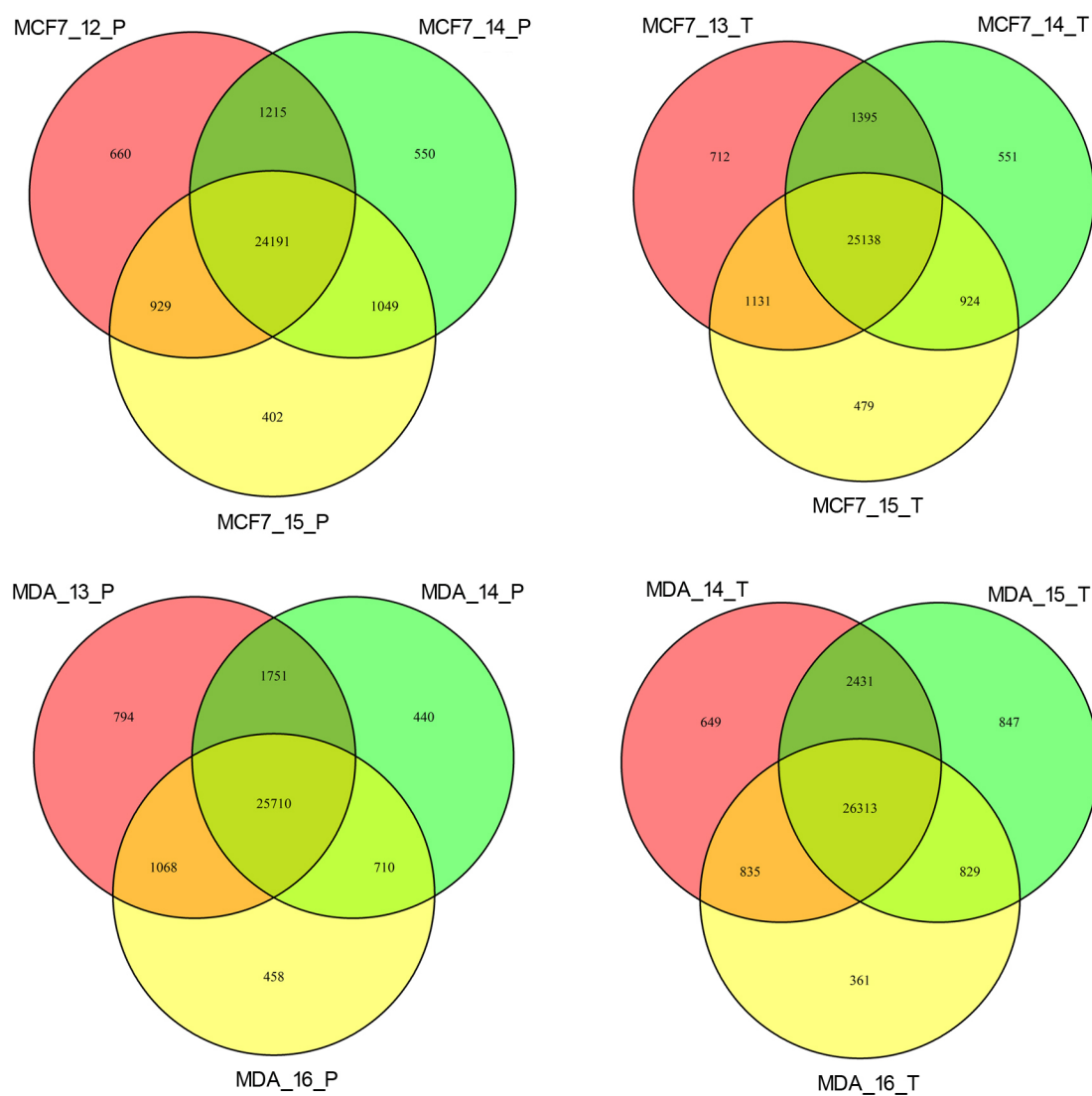

### lncRNA PCA

B.

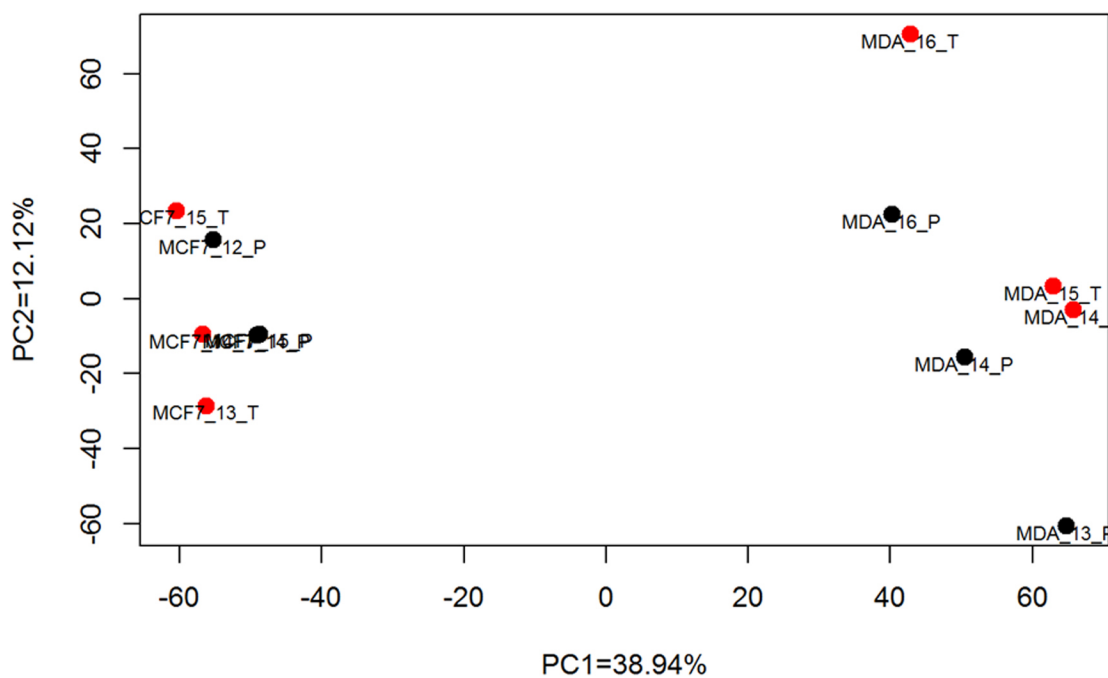

Supplement: Supplementary file 5 — Dataset 4 [file 41598_2018_36231_MOESM5_ESM.pdf]
